# Pollen Morphology of Selected Endemic Eudicots From the FLORAS Botanical Garden, Southern Bahia, Brazil

**DOI:** 10.1002/jemt.70005

**Published:** 2025-07-02

**Authors:** Agatha Carvalho Pinto, Amanda Spiller, Giovanna Candido, Jorge Antonio Silva Costa, Cristiana Barros Nascimento Costa, Mário Marques da Silva Junior, Jaílson Santos de Novais

**Affiliations:** ^1^ Postgraduate Program in Environmental Sciences and Technologies Bahia Federal Institute and Southern Bahia Federal University Porto Seguro Bahia Brazil; ^2^ Center for Environmental Sciences Training Southern Bahia Federal University Porto Seguro Bahia Brazil; ^3^ Institute of Humanities, Arts and Sciences at Sosígenes Costa Campus, Southern Bahia Federal University Porto Seguro Bahia Brazil

**Keywords:** Atlantic forest, flora of Brazil, palynology, palynotaxonomy, pollen flora

## Abstract

The Atlantic Forest is home to about 40% of Brazil's flora. Approximately 50% of the 17,975 species occurring in this biome are endemic, and many are threatened with extinction, such as brazilwood, *Paubrasilia echinata* (Lam.) Gagnon, H.C.Lima, & G.P.Lewis. Despite such importance, studies to characterize the pollen morphology of this rich flora have been unevenly distributed throughout this phytogeographic domain. Therefore, we aimed to describe the pollen morphology of 12 selected endemic species of eudicots from the FLORAS Botanical Garden, a remnant of the Atlantic Forest at intermediate stages of natural regeneration in southern Bahia State, Brazil. The analyzed species were *Andira fraxinifolia* Benth., *Cranocarpus mezii* Taub., *Desmodium juruenense* Hoehne, *Eschweilera ovata* (Cambess.) Mart. ex Miers, *Himatanthus bracteatus* (A.DC.) Woodson, *Lantana undulata* Schrank, *Lundia longa* (Vell.) DC., *Mimosa caesalpiniifolia* Benth., *Ouratea cuspidata* (A.St.‐Hil.) Engl., *Paubrasilia echinata* (Lam.) Gagnon, H.C.Lima & G.P.Lewis, *Solanum polytrichum* Moric., and *Stigmaphyllon blanchetii* C.E.Anderson. Flower buds were collected from exsiccatae of the GCPP herbarium. The pollen material was acetolyzed, measured, described, and photodigitalized using optical microscopy. Pollen grains were recorded in monads or polyads; small to large sized; spherical, prolate‐spheroidal, subprolate, or prolate; apolar or isopolar; with triangular, subtriangular, subcircular, or circular amb; 3‐colpate, 3‐colporate or pantoporate; and with psilate, (micro)reticulate, granulate, or scabrate exine. The descriptions revealed the local morphopalynological diversity, contributing to the understanding of the pollen florula of the FLORAS Botanical Garden and the Central Atlantic Forest Ecological Corridor.

## Graphical abstract

This study describes the pollen morphology of twelve endemic species from the Atlantic Forest, revealing subtle morphopollinic variations. Diagnostic characters such as pollen shape, exine ornamentation, and amb type highlight the region's floristic diversity, providing valuable insights into the ecological and conservation significance of Brazil's threatened biodiversity.

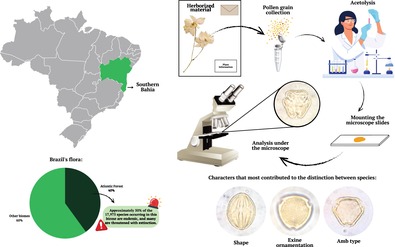

**Summary**

- The endemic species studied differ palynologically.
- The morphopollinic characters varied subtly among the species.
- Pollen shape, exine ornamentation and amb stood out as diagnostic characters.

## 1 Introduction

The Atlantic Forest is home to about 40% of Brazil's flora (Flora e Funga do Brasil 2024). This representativeness indicates that the biome has proportionally greater biological diversity than the Amazon when considering territorial extent. However, despite its relevance, the biome is facing severe degradation, threatening regional biological diversity. The Atlantic Forest originally spanned 17 Brazilian states, but today it has only 12.4% of mature forests, which house the largest and best preserved remnants, as well as the largest carbon stocks and the greatest biodiversity (Fundação SOS Mata Atlântica and Instituto Nacional de Pesquisas Espaciais 2024).

According to a survey conducted by Flora e Funga do Brasil (2024), the Atlantic Forest currently comprises 17,975 plant species belonging to 380 families and 2615 genera. Of the recorded vascular plants, about 50% are endemic and many are threatened with extinction. Such is the case of brazilwood, *Paubrasilia echinata* (Lam.) Gagnon, H.C.Lima, & G.P.Lewis (BFG‐Leguminosae 2024), which is considered the national symbol of the country. These keystone species are fundamental to ecosystem dynamics, as their extinction can lead to the disappearance of several other species that directly depend on them (Cardoso 2016).

The Atlantic Forest provides essential ecological services that support human life, such as preservation of soil integrity and fertility, protection of water bodies, pollination, thermal regulation, and carbon sequestration and storage. Accordingly, the Convention on Biological Diversity (1992) defined the in situ conservation of ecosystems and natural habitats as a central goal. However, only 10% of the Atlantic Forest vegetation is enclosed in protected areas and indigenous territories (Vancinea et al. 2024).

Botanical gardens are environments that promote *ex situ* biodiversity conservation through scientific collections. For instance, the FLORAS Botanical Garden (FLORAS BG), one of the most recent botanical gardens created in Brazil, fulfills this valuable ecological role. The initiative was funded in 2018 on the Sosígenes Costa Campus of the Federal University of Southern Bahia (USFB), in the city of Porto Seguro. The FLORAS BG comprises 232,000 m^2^, with approximately 20,000 m^2^ of built area, and includes an important remnant of the Atlantic Forest in intermediate stages of natural regeneration since the year 2000 (Antunes et al. 2020; Pinto et al. 2019).

The FLORAS BG lies within the Central Atlantic Forest Ecological Corridor, which encompasses southern Bahia, Espírito Santo, and a small region to the northeast of Minas Gerais State. Ecological corridors serve as locations where threatened and restricted species can be conserved (Silva et al. 2016), in contrast to the extensive monoculture in southern Bahia, such as the eucalyptus plantations managed by pulp and paper industries. However, the main scenario that directly or indirectly interferes with the FLORAS BG is its proximity to farms and the rapidly expanding urban area of the municipality of Porto Seguro.

Previous floristic surveys recorded more than 279 species in the FLORAS BG, highlighting the families Fabaceae, Asteraceae, Malvaceae, Euphorbiaceae, Arecaceae, Asparagaceae, Bignoniaceae, and Orchidaceae (Antunes et al. 2020; Pinto et al. 2019). So far, the flora recorded for the garden includes 19 Brazilian endemic species of eudicots, underscoring the region's importance for preserving the Atlantic Forest flora (Antunes et al. 2020; Pinto et al. 2019).

Palynological studies of the Brazilian Atlantic Forest began in the 1960s (Barth and Bouzada 1964) and continued in the following decades. Most research efforts have focused on the states of Espírito Santo (Lorente et al. 2017) and São Paulo (Barth and Barbosa 1972; Corrêa et al. 2018; Fernandez et al. 2021; Melhem et al. 1984; Melo et al. 2017; Silva et al. 2014). Despite southern Bahia's recognition as a hotspot of plant diversity (Brasil, 2006; Rezende et al. 2018) with high rates of endemism, there is a notable lack of studies on the pollen morphology of its rich flora (Pinto and Novais 2023). Some as yet nonsystematic studies have palynologically analyzed species from *restingas* on the State's northern coast (Santana‐Souza et al. 2024), or worked with applied palynology, especially melissopalynology (Alves and Santos 2019; Araújo and Novais 2023; Bandeira and Novais 2020, 2021; Dórea et al. 2010; Matos and Santos 2017a, 2017b, 2019).

Information on pollen morphology is essential for applied fields of research, such as melissopalynology, as it enables the correct identification of pollen types in bee products, such as honey and bee pollen. Southern Bahia offers great potential for beekeeping and meliponiculture. However, melissopalynological studies in this region reported challenges in identifying pollen types in honey and other products due to a lack of reference catalogs (Araújo and Novais 2023; Bandeira and Novais 2020, 2021). In view of these limitations, this study aimed to contribute to the knowledge of the pollen flora of the southern Bahia Atlantic Forest by characterizing the pollen morphology of some endemic species of eudicots occurring in vegetation remnants in the FLORAS Botanical Garden.

## 2 Material and Methods

### 2.1 Study Area

**FIGURE 1:**
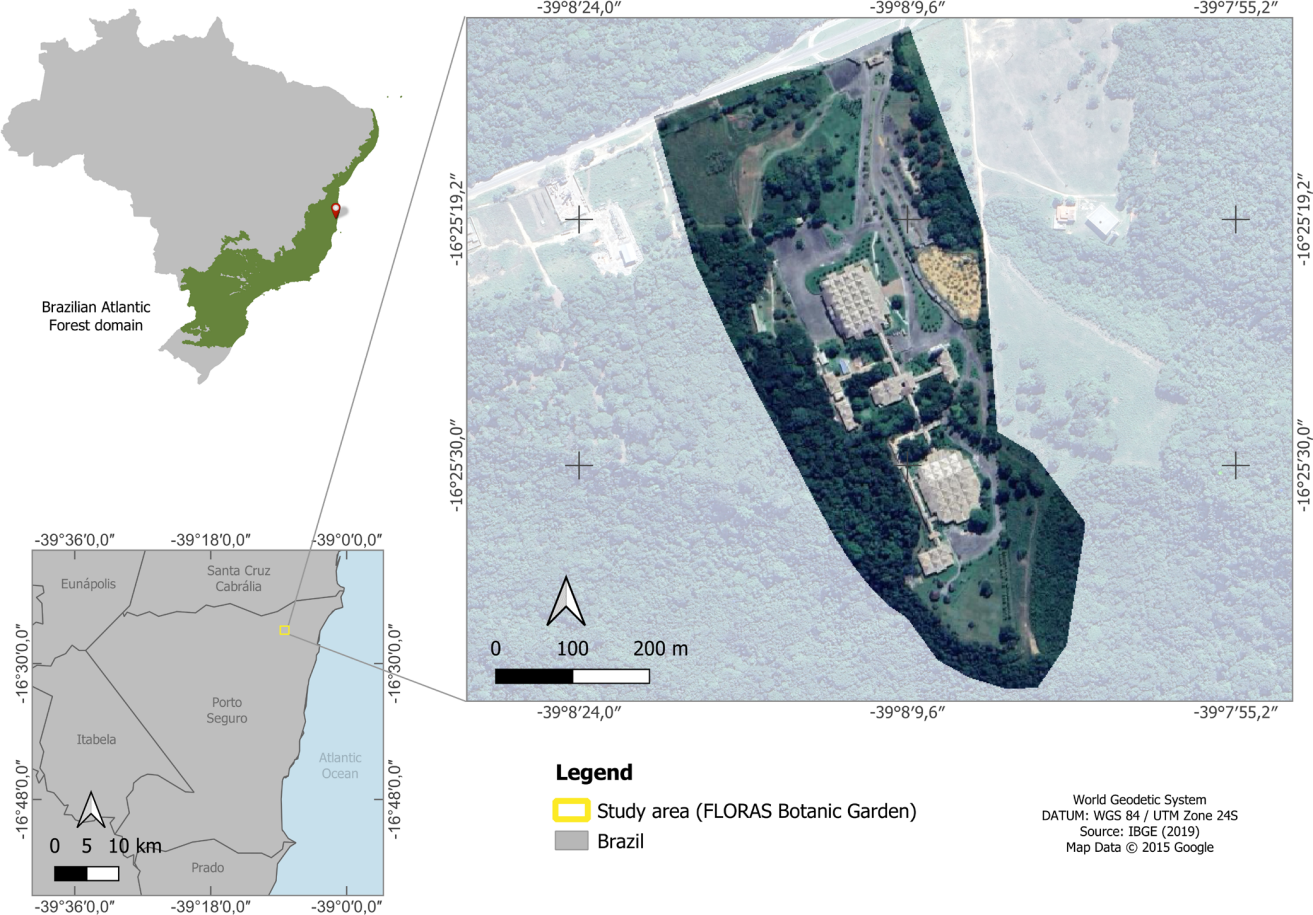
Map showing the Atlantic Forest fragment on the Sosígenes Costa Campus, Federal University of Southern Bahia, Brazil, with the FLORAS Botanical Garden highlighted.

The FLORAS Botanical Garden is situated on the Sosígenes Costa *Campus* at UFSB, Porto Seguro, Bahia State, Brazil (16°25′22.90″ S 39°08′11.56″ W, circa 75 m a.s.l.). The botanical garden has a total area of 230,000 m^2^, with a built‐up area of approximately 20,000 m^2^, which includes the Professor Geraldo C. P. Pinto (GCPP) herbarium (Figure 1). The garden encompasses an Atlantic Forest fragment at intermediate stages of regeneration. The vegetation is classified as lowland dense ombrophilous forest. The climate is of the Af type, that is, rainy, hot, and humid, with no defined dry season. The average temperature ranges from 19°C to 29°C and the monthly rainfall from 78 to 154 mm (Pinto et al. 2019).

Bahia spans over 564,700 km^2^ (Instituto Brasileiro de Geografia e Estatística, 2024), ranking fifth in territorial extent among Brazilian states. Despite its vast territory, it only has the Salvador Botanical Garden and the FLORAS Botanical Garden, only the latter of which is affiliated to the Brazilian Network of Botanical Gardens.

### 2.2 Collection of Pollen Material

The pollen material was obtained directly from exsiccatae and duplicate specimens of the GCPP herbarium. These specimens were collected by Pinto et al. (2019) and Antunes et al. (2020) between 2015 and 2018 along preexisting trails of the forest fragment and ornamental gardens at the FLORAS Botanical Garden. In addition, field trips complemented collections with little polliniferous material, if the species were in bloom.

Collections were focused on closed and well‐developed flower buds, the former to avoid contamination by pollen grains of other species and the latter to afford mature pollen grains. Pollen grains were extracted from flower buds of 12 endemic species available in the GCPP collection: *Andira fraxinifolia* Benth. (Fabaceae), *Cranocarpus mezii* Taub. (Fabaceae), *Desmodium juruenense* Hoehne (Fabaceae), *Eschweilera ovata* (Cambess.) Mart. ex Miers (Lecythidaceae), *Himatanthus bracteatus* (A.DC.) Woodson (Apocynaceae), *Lantana undulata* Schrank (Verbenaceae), *Lundia longa* (Vell.) DC. (Bignoniaceae), *Mimosa caesalpiniifolia* Bent. (Fabaceae), *Ouratea cuspidata* (A.St.‐Hil.) Engl. (Ochnaceae), *Paubrasilia echinata* (Lam.) Gagnon, H.C.Lima & G.P.Lewis (Fabaceae), *Solanum polytrichum* Moric. (Solanaceae), and *Stigmaphyllon blanchetii* C.E.Anderson (Malpighiaceae). Seven other endemic species of eudicots recorded in the FLORAS Botanical Garden were excluded from this study because of a lack of fertile material for analysis.

### 2.3 Laboratory Procedures and Analysis

Pollen samples were acetolyzed according to Erdtman (1952, 1960). The resulting pollen sediment was mounted in glycerinated gelatin under a coverslip on a microscope slide and sealed with paraffin (Salgado‐Labouriau 1973). Microscopic analysis, descriptions, and measurements were performed within 7 days of slide preparation. Each species was photomicrographed using a camera coupled to a Motic Panthera U trinocular microscope. Images were acquired using Motic Images Plus 3.0 software. The slides were included in the palinoFLORAS microscope slide collection of the FLORAS Botanical Garden (Novais et al. 2018).

Pollen grains were measured according to standard procedures, as recommended in the FLORAS pollen florula protocol (Pinto and Novais 2023). The following variables were analyzed: polar axis (P), equatorial diameter (E), equatorial diameter in polar view (Ep), sexine thickness, and nexine thickness. A total of 25 pollen grains were measured for P, E, and Ep, and 10 pollen grains were measured for exine thickness per species. Subsequently, the arithmetic mean and standard deviation of the mean were calculated.

Descriptions of dispersion unit, pollen size, shape, polarity, symmetry, amb, apertures, exine, and measurements follow a reference work in the field (Punt et al. 2007). Additionally, the descriptions of each species include the examined specimen, notes on pollen morphology, phytogeographic information (Flora e Funga do Brasil 2024), and conservation status, when available (IUCN 2024).

## 3 Results and Discussion

### 3.1 Apocynaceae

**FIGURE 2:**
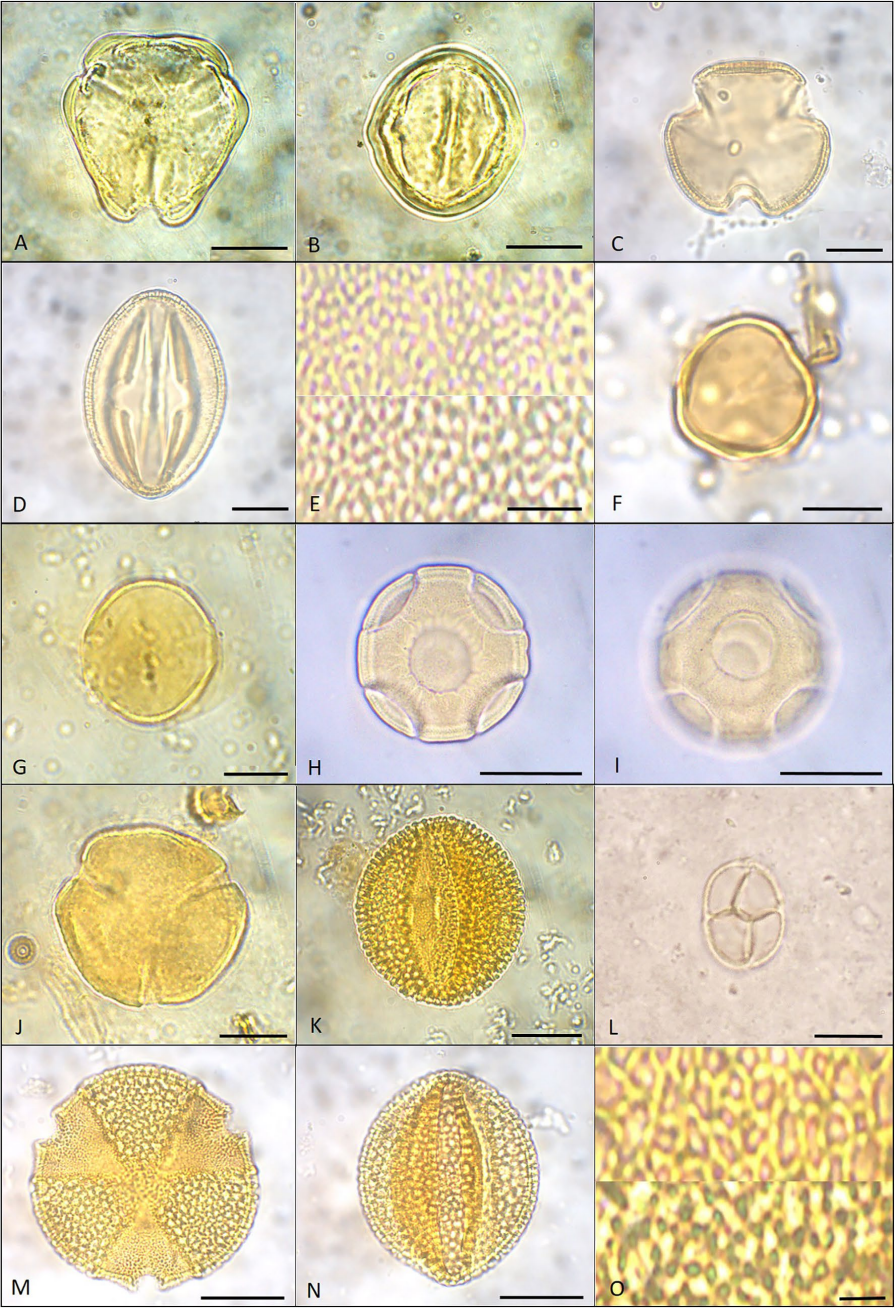
Photomicrographs of pollen grains of endemic species of eudicots sampled at the FLORAS Botanical Garden. (A, B) *Himatanthus bracteatus*: (A) polar view, optical section; (B) equatorial view, optical section. (C–E) *Lundia longa*: (C) polar view, optical section; (D) equatorial view, optical section; (E) LO–analysis. (F, G) *Andira fraxinifolia*: (F) polar view, optical section; (G) equatorial view, optical section. (H, I) *Cranocarpus mezii*: (H) optical section; (I) equatorial view, surface detail. (J, K) *Desmodium juruenense*: (J) polar view, optical section; (K) equatorial view, optical section. (L) *Mimosa caesalpiniifolia*: Equatorial view, optical section. (M–O) *Paubrasilia echinata*: (M) polar view, optical section; (N) equatorial view, optical section; (O) LO–analysis. Scale bars: A–E, H, I, M–O = 20 μm; F, G, J–L = 10 μm.

*Himatanthus bracteatus* (A.DC.) Woodson (Figure 2A,B).

Pollen grains in monads, medium‐sized, subprolate, isopolar, radially symmetric, triangular amb, 3‐colporate, angulaperturate, with psilate exine and sexine thicker than nexin.

**Measurements (μm):** *P* = 45.0 ± 7.07 (40.0–50.0), *E* = 36.66 ± 5.77 (30.0–40.0), *Ep* = 41.95 ± 2.45 (38.0–47.0), sexine thickness = 1.12, nexine thickness = 0.82.

**Specimen examined:** BRAZIL, Bahia: Porto Seguro, BR‐367, UFSB, Sosígenes Costa Campus, March 31st, 2023, J.S. Novais 148 (GCPP 00541, palinoFLORAS R0119).

**Notes:** The observed features agree with those reported by Rodrigues et al. (2017), except for suboblate grains. Of note, Rodrigues et al. (2017) reported higher values of exine thickness than those observed here, particularly for the sexine.

According to the Flora e Funga do Brasil (2024), *H. bracteatu*s is a terrestrial tree with recorded occurrence in the Atlantic Forest phytogeographic domain, found in deciduous forests in the states of Alagoas, Bahia, Espírito Santo, Minas Gerais, Paraíba, Pernambuco, Rio de Janeiro, Rio Grande do Norte, and Sergipe. The conservation status of the species is listed as least concern (LC) (IUCN 2024).

### 3.2 Bignoniaceae

*Lundia longa* (Vell.) DC. (Figure 2C–E).

Pollen grains in monads, large, subprolate, isopolar, radially symmetric, triangular amb, 3‐colporate, angulaperturate, with microreticulate homobrochate exine and sexine thicker than nexin.

**Measurements (μm):** *P* = 55.0 ± 3.2 (50.0–61.0), *E* = 43.62 ± 2.27 (39.0–47.0), *Ep* = 34.88 ± 5.15 (30.0–48.0), sexine thickness = 1.6, nexine thickness = 1.41.

**Specimen examined:** BRAZIL, Bahia: Porto Seguro, BR‐367, UFSB, Sosígenes Costa Campus, May 19th, 2017, V. C. Santos 73 (GCPP 00511, palinoFLORAS R0087).

**Notes:** The observed characters are similar to those presented by Lorente et al. (2017) and Ybert et al. (2017) for other species of the genus (*Lundia virginalis* DC. and *L. cordata* DC.), mainly with regard to pollen shape, amb, and aperture number and type.

According to the Flora e Funga do Brasil (2024), *L. longa* is a terrestrial liana/creeper native and endemic to Brazil. It occurs in the states of Alagoas, Bahia, Ceará, Espírito Santo, Minas Gerais, Paraíba, Pernambuco, Rio de Janeiro, Rio Grande do Norte, São Paulo, and Sergipe in areas of riparian or gallery forests, ombrophilous forests (rainforests), and *restingas* (coastal forest). There are no records of the conservation status of this species (IUCN 2024).

### 3.3 Fabaceae

*Andira fraxinifolia* Benth. (Figure 2F,G).

Pollen grains in monads, small, prolate, isopolar, radially symmetric, subcircular amb, 3‐colporate, with microreticulate exine, sexin, and nexin difficult to distinguish.

**Measurements (μm):** *P* = 23.12 ± 1.45 (21.0–26.0), *E* = 13.87 ± 2.77 (5.0–17.0), *Ep* = 19.5 ± 0.70 (19.0–20.0), exine thickness = 1.1.

**Specimen examined:** BRAZIL, Bahia: Porto Seguro, BR‐367, UFSB, Sosígenes Costa Campus, October 1st, 2015, A.C. Pinto et al. 03 (GCPP 00027, palinoFLORAS R0073).

**Notes:** Luz et al. (2013) described the species as having characteristics similar to those recorded here, except for its prolate‐spheroidal shape. Ybert et al. (2017) also described the species with some differing characteristics, such as a triangular amb and psilate exine.

The Flora e Funga do Brasil (2024) describes *A. fraxinifolia* as a rupicolous tree, occurring in anthropic areas of riparian or gallery forests, semideciduous seasonal forests, ombrophilous forests (rainforests), and *restingas*. The species has been recorded in the following states: Alagoas, Bahia, Ceará, Federal District, Espírito Santo, Goiás, Mato Grosso do Sul, Minas Gerais, Paraíba, Paraná, Pernambuco, Piauí, Rio de Janeiro, Rio Grande do Norte, Santa Catarina, São Paulo, and Sergipe. *A. fraxinifolia* is often confused with *Andira anthelmia*, *A. ormosioides*, and *A. legalis* in tropical forests and *restingas* along the Brazilian Atlantic coast. Flower size is the main character used to distinguish the species. Its conservation status is LC; however, the conservation data are from 2012, requiring an update (IUCN 2024).

*Cranocarpus mezii* Taub. (Figure 2H,I).

Pollen grains in monads, medium‐sized, apolar, 4‐8‐pantoporate, operculate pores, with scabrate exine, and sexine thicker than nexine.

**Measurements (μm):** *D*_1_ = 44.54 ± 3.0 (40.15–49.11), *D*_2_ = 40.61 ± 1.55 (38.06–42.56), sexine thickness = 1.59, nexine thickness = 0.95, pore diameter = 12.75.

**Specimen examined:** BRAZIL, Bahia: Porto Seguro, Residencial Outeiro da Glória, Quadra 17, February 24th, 2025, J. A. S. Costa 2.513 (GCPP 3272, palinoFLORAS R0032).

**Notes:** We found no previous studies describing the pollen morphology of *C. mezii*. However, within the genus, Ferguson and Skvarl (1979) described the pollen grains of *C. martii* Benth. as also being spheroidal, porate, and with the presence of operculum. However, this species has a greater number of pores (15–24) than *C. mezii*.

According to Flora e Funga do Brasil (2024), *C. mezii* is a terrestrial shrub or subshrub, native and endemic to Brazil. It occurs in the Atlantic Forest phytogeographic domain in ombrophilous forest and *restinga* vegetation. There are no records of the conservation status of this species (IUCN 2024).

*Desmodium juruenense* Hoehne (Figure 2J,K).

Pollen grains in monads, medium‐sized, prolate, isopolar, radially symmetric, subtriangular amb, 3‐colpate, angulaperturate, with reticulate exine, and sexine slightly thicker than nexine.

**Measurements (μm):** *P* = 38.14 ± 2.67 (34.0–42.0), *E* = 28.0 ± 5.06 (24.0–36.0), *Ep* = 35.58 ± 4.12 (31.0–47.0), sexine thickness = 1.1, nexine thickness = 0.9.

**Specimen examined:** BRAZIL, Bahia: Porto Seguro, BR‐367, UFSB, Sosígenes Costa Campus, May 17th, 2016, A.C. Pinto et al. 32 (GCPP 00063, palinoFLORAS R0071).

**Notes:** The material described here is in agreement with palynological descriptions of the genus, such as in Lorente et al. (2017) and Ybert et al. (2017). The cited studies described *Desmodium axillare* (Sw.) DC., *D. tortuosum* (Sw.) DC., and *D. adscendens* (Sw.) DC. These species are similar with regard to pollen size and aperture type and number.

According to the Flora e Funga do Brasil (2024), *D. juruenense* is a terrestrial subshrub with a confirmed geographical distribution in the states of Amazonas, Goiás, Mato Grosso, and Rondônia. The species occurs in the following vegetation types: Amazonian *campinaranas*, várzea forests, Cerrado *s.l*., and riparian or gallery forests. Thus, the current record of the species is unusual, as it was sampled in an ombrophilous forest in Bahia State. There are no records of the conservation status of this species on the IUCN Red List (2024).

*Mimosa caesalpiniifolia* Benth. (Figure 2L).

Polyads with eight pollen grains each (ditetrads), arranged in two tetragonal (or less often rhomboidal or tetrahedral) tetrads, small‐sized, apertures not visualized, psilate to scabrate exine, sexine and nexin indistinct under optical microscopy.

**Measurements (μm):** *P* = 14.85 ± 0.90 (12.36–17.06), *E* = 11.84 ± 1.28 (10.49–14.44), exine thickness = 1.28.

**Specimen examined:** BRAZIL, Bahia: Porto Seguro, BR‐367, UFSB, Sosígenes Costa Campus, February 18th, 2025, A. Spiller 29 (GCPP 3273, palinoFLORAS R0033).

**Notes:** Lima et al. (2008) described the pollen grains of *M. caesalpiniifolia* in the *Mimosa caesalpiniifolia* pollen type, which includes species with small, psilate pollen grains, in polyads with 8 grains (ditetrads) arranged in tetragonal, decussate or tetrahedral tetrads.

*M. caesalpiniifolia* comprises terrestrial shrubs and trees, native and endemic to Brazil (Flora e Funga do Brasil 2024). It is found mainly in the *Caatinga* domain, in *caatinga s. s*. vegetation, riverine and/or gallery forests, and anthrophic areas. Its conservation status is LC (IUCN 2024).

*Paubrasilia echinata* (Lam.) Gagnon, H.C.Lima & G.P.Lewis (Figure 2M–O).

Pollen grains in monads, large‐sized, subprolate, isopolar, radially symmetric, circular amb, 3‐colporate, with reticulate, heterobrochate exine, and sexine thicker than nexine.

**Measurements (μm):** *P* = 51.66 ± 2.79 (48.0–57.0), *E* = 41.2 ± 3.54 (36.0–46.0), *Ep* = 55.4 ± 2.98 (50.0–60.0), sexine thickness = 3.0, nexine thickness = 1.0.

**Specimen examined:** BRAZIL, Bahia: Porto Seguro, BR‐367, UFSB, Sosígenes Costa Campus, February 22th, 2019, A.C. Pinto et al. s/n (GCPP 00501, palinoFLORAS R0123).

**Notes:** The pollen morphology of *P. echinata* (formerly *Caesalpinia echinata* ) has been described by authors such as Corrêa (2003), Antonio‐Domingues et al. (2018), and Pinto and Novais (2023), whose information coincides with that presented here.

According to Flora e Funga do Brasil (2024), *P. echinata* corresponds to terrestrial trees that are native and endemic to Brazil. It occurs in the Atlantic Rainforest Domain, in seasonally semideciduous forest vegetation, ombrophilous forest, and *restingas*. According to IUCN (2024), the conservation status of the species is EN.

### 3.4 Lecythidaceae

**FIGURE 3:**
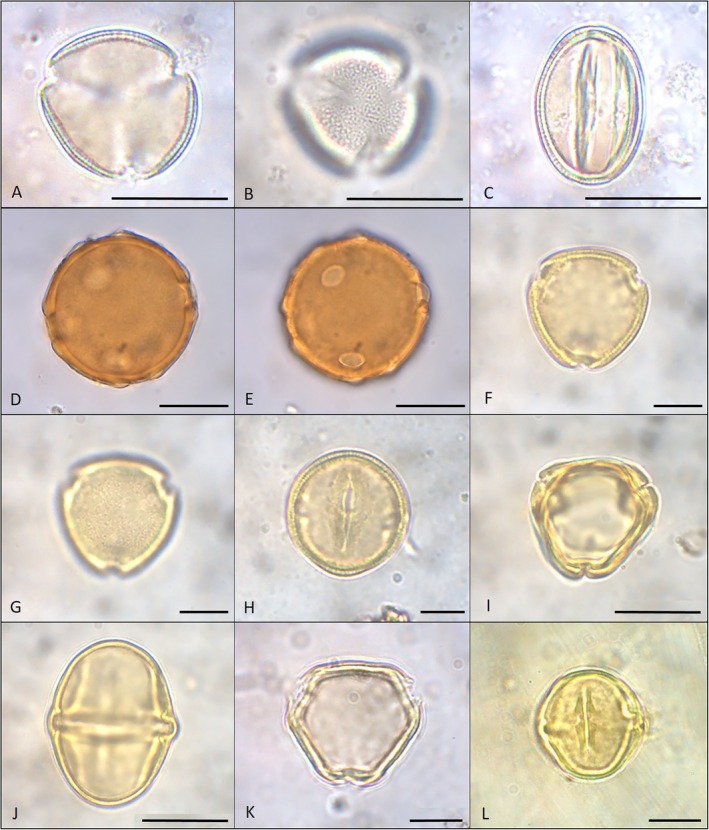
Photomicrographs of pollen grains of endemic species of eudicots sampled at the FLORAS Botanical Garden. (A–C) *Eschweilera ovata:* (A) polar view, optical section; (B) polar view, surface detail; (C) equatorial view, optical section. (D, E) *Stigmaphyllon blanchetii:* (D) optical section; (E) equatorial view, surface detail. (F–H) *Ouratea cuspidata*: (F) polar view, optical section; (G) polar view, surface detail; (H), equatorial view, optical section. (I, J) *Solanum polytrichum* : (I) polar view, optical section; (G) equatorial view, optical section. (K–L) *Lantana undulata*: (A) polar view, optical section; (B) equatorial view, optical section. Scale bars: A–E, I, J = 20 μm; F–H, K, L = 10 μm.

*Eschweilera ovata* (Cambess.) Mart. ex Miers (Figure 3A–C).

Pollen grains in monads, medium‐sized, prolate, isopolar, radially symmetric, subtriangular amb, 3‐colporate, angulaperturate, with microreticulate exine and sexine slightly thicker than nexine.

**Measurements (μm):** *P* = 27.33 ± 1.83 (24.0–30.0), *E* = 19.4 ± 2.16 (18.0–23.0), *Ep* = 25.1 ± 2.02 (21.0–28.0), sexine thickness = 0.87, nexine thickness = 0.76.

**Specimens examined:** BRAZIL, Bahia: Porto Seguro, BR‐367, UFSB, Sosígenes Costa Campus, February 11th, 2017, V.C. Santos et al. 09 (GCPP 00194, palinoFLORAS R0106).

**Notes:** This species was described by Lorente et al. (2017) and Ybert et al. (2018). The former study reported some different characters from those observed here, such as small pollen size and circular shape. The latter study differed from the current one with regard to the scrobiculate (=perforate) exine and triangular amb.

*E. ovata* is a terrestrial tree found in ombrophilous forests (rainforests) and *restingas*. It has wide geographical distribution, encompassing the states of Alagoas, Amapá, Bahia, Ceará, Espírito Santo, Maranhão, Mato Grosso, Minas Gerais, Pará, Paraíba, Pernambuco, Piauí, Rio Grande do Norte, and Sergipe (Flora e Funga do Brasil 2024). According to IUCN (2024), the conservation status of the species is LC.

### 3.5 Malpighiaceae

*Stigmaphyllon blanchetii* C.E.Anderson (Figure 3D,E).

Pollen grains in monads, large, apolar, 2‐9‐porate, with granulate exine and sexine thicker than nexine.

**Measurements (μm):** *D*_1_ = 55.8 ± 5.76 (40.0–65.0), *D*_2_ = 55.8 ± 6.39 (37.0–68.0), sexine thickness = 2.51, nexine thickness = 1.33, pore diameter = 5.23.

**Specimen examined:** BRAZIL, Bahia: Porto Seguro, BR‐367, UFSB, Sosígenes Costa Campus, October 1st, 2015, C.B.N. Costa et al. 507A (GCPP 00028, palinoFLORAS R0089).

**Notes:** The descriptions agree with those reported in the palynological literature for the genus, as in Lorente et al. (2017), who described *Stigmaphyllon paralias* A.Juss., and in Gonçalves‐Esteves et al. (2007) and Belonsi and Gasparino (2015), who described *Stigmaphyllon lalandianum* A.Juss., *S. arenicola* C.E.Anderson, *S. auriculatum* (Cav.) A.Juss., *S. ciliatum* (Lam.) A.Juss., and *S. paralias* . These species were reported to have pollen with granulate exine or granulate areas.

According to the Flora e Funga do Brasil (2024), *S*. *blanchetii* is a terrestrial liana/creeper occurring in Alagoas, Bahia, Espírito Santo, Minas Gerais, Paraíba, Pernambuco, Rio Grande do Norte, and Sergipe States in semideciduous seasonal forests, dense ombrophilous forests, and *restingas*. There are no records of its conservation status on the IUCN Red List (2024).

### 3.6 Ochnaceae

*Ouratea cuspidata* (A.St.‐Hil.) Engl. (Figure 3F–H).

Pollen grains in monads, small, prolate‐spheroidal, isopolar, radially symmetric, triangular amb, 3‐colporate, angulaperturate, with scabrate exine and sexine slightly thicker than nexine.

**Measurements (μm):** *P* = 22.71 ± 1.79 (20.0–25.0), *E* = 22.28 ± 2.42 (19.0–25.0), *Ep* = 21.83 ± 1.46 (19.0–24.0), sexine thickness = 0.82, nexin thickness = 0.80.

**Specimen examined:** BRAZIL, Bahia: Porto Seguro, BR‐367, UFSB, Sosígenes Costa Campus, October 27th, 2016, A.C. Pinto et al. 41 (GCPP 00112, palinoFLORAS R0092).

**Notes:** The pollen grains of the species were described by Lorente et al. (2017), with many similarities to those of the current study, except for the suboblate shape. The species was also described by Salgado‐Labouriau (1973) and Ybert et al. (2018). The cited studies reported similar measurements but a microgranulose exine (Ybert et al. 2018) and oblate‐spheroidal shape (Salgado‐Labouriau 1973).

According to the Flora e Funga do Brasil (2024), *O. cuspidata* is a terrestrial tree distributed across Bahia, Ceará, Espírito Santo, Paraíba, Rio de Janeiro, Rio Grande do Norte, São Paulo, and Sergipe States. It occurs in ombrophilous forests (rainforests) and *restingas*. There is no information about its conservation status on the IUCN Red List (2024).

### 3.7 Solanaceae

*Solanum polytrichum* Moric. (Figure 3I,J).

Pollen grains in monads, medium‐sized, subprolate, isopolar, radially symmetric, subtriangular amb, 3‐colporate, angulaperturate, with psilate exine and sexine thicker than nexine.

**Measurements (μm):** *P* = 34.0 ± 3.4 (27.0–39.0), *E* = 28.5 ± 2.43 (23.0–31.0), *Ep* = 29.5 ± 2.12 (28.0–31.0), sexine thickness = 0.78, nexine thickness = 0.65.

**Specimen examined:** BRAZIL, Bahia: Porto Seguro, BR‐367, UFSB, Sosígenes Costa Campus, February 16th, 2017, A.C. Pinto et al. 47 (GCPP 00151, palinoFLORAS R0093).

**Notes:** The observations made in this study differ from those presented by Lorente et al. (2017), who recorded suboblate to circular pollen with scabrate exine.

According to the Flora e Funga do Brasil (2024), *S. polytrichum* is a terrestrial shrub distributed across Alagoas, Bahia, Espírito Santo, Minas Gerais, Paraíba, Piauí, Rio de Janeiro, and Sergipe States. It is found in the following vegetation types: deciduous seasonal forests, semideciduous seasonal forests, ombrophilous forests, and *restingas*. There are no records of its conservation status on the IUCN Red List (2024).

### 3.8 Verbenaceae

*Lantana undulata* Schrank (Figure 3K,L).

Pollen grains in monads, medium‐sized, prolate‐spheroidal, isopolar, radially symmetric, triangular amb, 3‐colporate, angulaperturate, with scabrate exine and sexine teacher than nexine.

**Measurements (μm):** *P* = 26.6 ± 2.65 (24.0–30.0), *E* = 25.66 ± 1.03 (24.0–27.0), *Ep* = 24.05 ± 1.16 (21.0–25.0), sexine thickness = 0.89, nexine thickness = 0.75.

**Specimen examined:** BRAZIL, Bahia: Porto Seguro, BR‐367, UFSB, Sosígenes Costa Campus, May 17th, 2016, A.C. Pinto et al. 2129 (GCPP 00075, palinoFLORAS R0094).

**Notes:** The species was described by Lorente et al. (2017) as having similar characters, except for small size and subprolate shape. The size and shape of pollen grains were similar to those described by Schroeder et al. (2019).

As described by the Flora e Funga do Brasil (2024), *L. undulata* is a terrestrial liana/creeper distributed in Alagoas, Bahia, Ceará, Espírito Santo, Minas Gerais, Paraíba, Pernambuco, Rio de Janeiro, Rio Grande do Norte, São Paulo, and Sergipe States. It occurs in riparian or gallery forests, ombrophilous forests, and *restingas*. There are no records of its conservation status (IUCN 2024).

## 4 Final Considerations

This study described the pollen characters of 12 endemic species of eudicots occurring in the FLORAS Botanical Garden. Pollen shape, exine ornamentation, and amb type contributed the most to the distinction between species. Nevertheless, such morphological differences were subtle, which highlights the need for additional studies to characterize the flora of the Atlantic Forest palynologically. The continuation of the study on the pollen morphology of endemic species of the Atlantic Forest from southern Bahia will contribute to applied studies in palynology, such as melissopalynology, in order to indicate the tropical species used by bees for honey production. For most of the species studied here, there is no information on their current conservation status.

## Author Contributions

**Agatha Carvalho Pinto:** conceptualization, investigation, writing – original draft, writing – review and editing, methodology, formal analysis, data curation, software, project administration. **Amanda Spiller:** investigation, writing – review and editing, visualization, methodology, formal analysis, software. **Giovanna Candido:** investigation, visualization, methodology, writing – review and editing, formal analysis, software. **Jorge Antonio Silva Costa:** writing – review and editing, validation, visualization. **Cristiana Barros Nascimento Costa:** validation, visualization, writing – review and editing. **Mário Marques da Silva Junior:** investigation, writing – review and editing, formal analysis, validation. **Jaílson Santos de Novais:** conceptualization, validation, visualization, funding acquisition, writing – review and editing, supervision, resources, methodology, formal analysis.

## Conflicts of Interest

The authors declare no conflicts of interest.

## Data Availability

The data that support the findings of this study are available from the corresponding author upon reasonable request.
